# Design of a Novel Series of Hetero-Binuclear Superhalogen Anions MM′X_4_^−^ (M = Li, Na; M′ = Be, Mg, Ca; X = Cl, Br)

**DOI:** 10.3389/fchem.2022.936936

**Published:** 2022-07-01

**Authors:** Hui Yang, Hui-Min He, Ning Li, Shang Jiang, Min-Jun Pang, Ying Li, Jian-Guo Zhao

**Affiliations:** ^1^ Institute of Carbon Materials Science, Shanxi Datong University, Datong, China; ^2^ Physics Department, Taiyuan Normal University, Taiyuan, China; ^3^ Institute of Theoretical Chemistry, College of Chemistry, Jilin University, Changchun, China

**Keywords:** superhalogen, chloride or bromine ligands, binuclear, vertical electron detachment energy, theoretical calculation

## Abstract

A series of hetero-binuclear superatom motifs involving chloride/bromide ligands, that is, MM′X_4_^−^ (M = Li, Na; M′ = Be, Mg, Ca; X = Cl, Br) anions, have been characterized by using many-body perturbation theory calculations. Large vertical electron detachment energies (VDEs, 5.470–6.799 eV) confirm the superhalogen identity of these anions. A larger VDE value can be obtained by introducing small M or large M′ central atoms and small halogen ligand atoms. Thus, one isomer of LiCaCl_4_^−^ possesses the largest VDE value. Besides, when the extra electron is shared by all ligand atoms or three bridging ligand atoms, the isomers have relatively larger VDE values.

## Introduction

Superhalogens are unusual molecules possessing higher electron affinity (EA) than those of any halogen atom (Gutsev and Boldyrev, 1981). They were first proposed by Gutsev and Boldyrev in 1981 and verified by a variety of theoretical chemical methods (Gutsev and Boldyrev, 1981). Meanwhile, a new class of highly stable anions (superhalogen anions) were also reported. Now, superhalogen anions have been proved to possess very large vertical electron detachment energies (VDEs) (Anusiewicz et al., 2003; Koirala et al., 2010; Yang et al., 2017; Li et al., 2019; Li et al., 2020; Zhao et al., 2020), even approaching 14 eV in certain systems (Freza and Skurski, 2010). Moreover, it is found that the superhalogen anions have much high stability, such as BF_4_^−^, AlCl_4_^−^, and AsF_6_^−^, and other superhalogen anions have been confirmed to be stable in crystalline solids or gaseous molecules.

In 1981, Gutsev and Boldyrev proposed the representative formula MX_k+1_ for a class of superhalogens, in which M represents the central main group atom, K is the highest valence of M atom, and X is the halogen atom (Gutsev and Boldyrev, 1981). In 1999, the superhalogen anions MX_2_^−^ (M = Li, Na; X = Cl, Br, and I) were reported by Wang et al., and their VDE values were experimentally measured for the first time and theoretically calculated applying the outer valence Green function (OVGF) method, which are consistent with each other well (Wang et al., 1999). Shortly afterward, the EA value of the BO_2_ superhalogen (Zhai et al., 2007) and the VDE value of the MX_3_^−^ (M = Be, Mg, Ca; X = Cl, Br) superhalogen anion (Elliott et al., 2005) were determined by the same experimental means. During subsequent studies on superhalogens and their corresponding anions, the central atom M of MX_k+1_ formula was no longer limited to the main group metal atoms (Anusiewicz et al., 2003; Elliott et al., 2005), and the transition metal atoms (Gutsev et al., 1999; Gutsev et al., 2001; Yang et al., 2003), coinage metal atoms (Feng et al., 2011; Lu et al., 2019), and nonmetal atoms (Arnold et al., 2002) could act as central atoms to construct superhalogens. In addition, the researchers found that increasing the number of central atoms benefits the dispersion of extra negative charges without increasing the repulsion between ligands. Therefore, some binuclear/multinuclear superhalogen anions have been proposed by experimental synthesis or theoretical prediction (Anusiewicz and Skurski, 2007; Anusiewicz, 2008; Czapla, 2017; Yang et al., 2017; Yang et al., 2018).

Besides, it is realized that halogen atoms are not the necessary units for the construction of superhalogens. In recent years, the ligands of superhalogens have been extended from halogen atoms to oxygen atoms (Gutsev et al., 1999; Zhai et al., 2007), acid functional groups (Anusiewicz, 2009b), various monovalent groups (Smuczynska and Skurski, 2009), nine-electron ligands (Sikorska et al., 2011), hydroxyl groups (Świerszcz and Anusiewicz, 2011), and electrophilic substituents (Anusiewicz, 2009a). In addition, M@Nk-type superhalogens with inclusion complexes of metal (Zhai et al., 2004) and carborane cage superhalogens (Pathak et al., 2011) have also been proposed. Recently, a new class of cluster was designed in which the central atom was modified by superhalogen ligands replacing the halogen ligands. These clusters have higher EAs than their superhalogen ligands; thus, they are termed “hyperhalogen" (Willis et al., 2010). Subsequently, many hyperhalogens with various geometries of superhalogen ligands have been proposed (Paduani et al., 2011; Gutsev et al., 2012; Sun et al., 2015; Sun et al., 2016; Yang et al., 2021; Dong et al., 2022).

Superhalogens play an important role in chemistry given the strong oxidation capability. For example, they can be used as capable oxidants to oxidize substances that have relatively high ionization potentials (e.g., O_2_ (Bartlett and Lohmann, 1962), noble gas atom (Bartlett, 1962), and (H_2_O)n clusters (Marchaj et al., 2013)). They can also be used to synthesize and prepare noble gas compounds (Saha et al., 2018; Chang et al., 2019), supersalts (Giri et al., 2014a), ion battery electrolytes (Giri et al., 2014b), ionic liquids (Srivastava et al., 2021), liquid crystalline molecules (Srivastava, 2021), solar cells (Kim et al., 2022), and so on. Therefore, exploring various new species classified as superhalogens and studying their structures, stability, and properties has become a significant and attractive research topic in recent years.

To the best of our knowledge, most hitherto proposed superhalogens are mono- or homo-nuclear. The hetero-nuclear superhalogens involving different central atoms, however, have received very little attention. The investigation on the influence of different ligands on hetero-nuclear superhalogen properties, however, has not been reported yet. In this research, we aim to design a new class of superhalogen anions with two different central atoms using chloride or bromine atoms as ligands. Consequently, the MM′X_4_^−^ (M = Li, Na; M′ = Be, Mg, Ca; X = Cl, Br) anions have been proposed and systematically investigated. The considerable VDE values of these anions confirm their superhalogen identity. The geometric features and relative stability of these anions were analyzed. Meanwhile, the correlations between their VDEs and structural features, ligand and central atoms, and extra electron distribution are also revealed. The present investigation predicts a new member of superhalogens and conduces to the development of new strong oxidizing agents.

## Computational Details

Initially, the structures of the MM′X_4_^−^ (M = Li, Na; M′ = Be, Mg, Ca; X = Cl, Br) anions were built by considering all the possible connection between M, M′, and X atoms. Then, all the constructed structures of anions were optimized using the Møller–Plesset perturbation method (MP2) (Møller and Plesset, 1934) together with the 6–311+G (3df) basis set (Yang et al., 2017; Yang et al., 2018). Meanwhile, frequency analysis was performed at the same computational level to ensure that the obtained structures are stable on potential energy surfaces without imaginary frequency. Natural bond orbital (NBO) (Reed et al., 1985) and single-point energy calculations were carried out at the same level.

The vertical electron detachment energies (VDEs) of the MM′X_4_^−^ anions were calculated applying the outer valence Green function (OVGF) approximation (Cederbaum, 1975) with the 6–311+G (3df) basis set. The smallest pole strength (PS) in our study is 0.90, justifying the validity of the OVGF method (Zakrzewski et al., 1996).

The above-mentioned calculations were performed using the GAUSSIAN 16 program package (Frisch et al., 2016). The plots of molecular structures and orbitals were generated with the GaussView program (Dennington et al., 2016).

## Results and Discussion

### Geometrical Structures and Relative Stability

**FIGURE 1:**
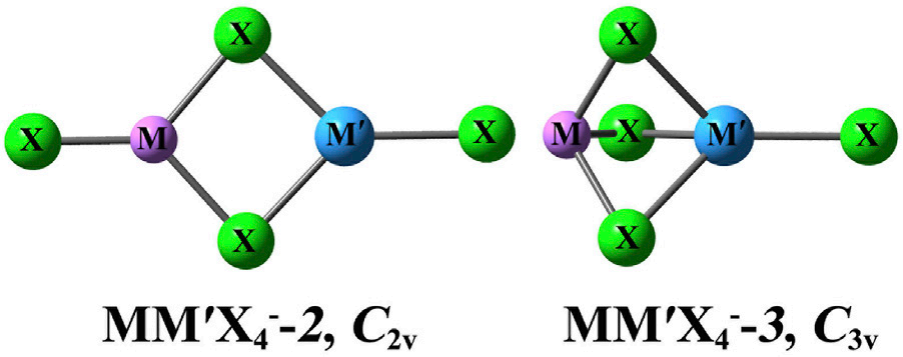
Optimized structures of the MM′X_4_^−^ anions at the MP2/6–311+G (3df) level. Color legend: green for X atom, light purple for M atom, and blue for M′ atom.

The optimized geometries of MM'X_4_^−^anions are depicted schematically in Figure 1. The relative energies, the lowest vibrational frequencies, bond lengths, and angles are listed in Tables 1, 2. As shown in the figure, each MM'X_4_^−^ anion has two types of structures, that is, central atoms M and M′ are connected by two or three bridging X atoms. Notably, these two structures are also presented in the superhalogen anions with F ligands (Yang et al., 2017). Unlike MM'F_4_^−^ anions (Yang et al., 2017), the structures involving one bridging ligand atom are not stable, which turn to the above two types of structures after optimization. In terms of the number of bridging X atoms, the isomers of MM'X_4_^−^ are termed MM'X_4_^−^-***2*** and MM'X_4_^−^-***3***, respectively. For the sake of convenience, the terminal X atoms that bind with M and M′ atoms are named X_*t*_ and X_*t*_’, respectively, and the bridging X atom that connects M and M′ atoms is named X_*b*_.

**TABLE 1:** Relative energies *E*_rel_ (kcal/mol), the lowest vibrational frequencies *υ* (cm^−1^), total NBO charges on the MCl subunit (|e|), vertical detachment energies VDE (eV), bond lengths (Å), and select bond angles (degree) of the MM′Cl_4_^−^ (M = Li, Na; M′ = Be, Mg, Ca) anions.

*Isomer*	Symmetry	*E*_rel_	*υ*	Q^a^	VDE	Cl_*t*_-M	M-Cl_*b*_	Cl_*b*_-M′	M′-Cl_*t*_'	∠Cl_*b*_M'Cl_*t*_'
LiBeCl_4_^−^-***2***	*C*_*2v*_	0.00	24	−0.073	6.240	2.146	2.427	1.940	1.888	123.6
LiBeCl_4_^−^-***3***	*C*_*3v*_	9.09	127	0.049	6.275		2.292	2.197	1.923	117.6
LiMgCl_4_^−^-***3***	*C*_*3v*_	0.00	86	−0.017	6.700		2.325	2.425	2.266	124.5
LiMgCl_4_^−^-***2***	*C*_*2v*_	4.48	21	−0.075	6.180	2.150	2.459	2.289	2.242	129.3
LiCaCl_4_^−^-***3***	*C*_*3v*_	0.00	65	−0.066	6.799		2.327	2.691	2.559	129.7
LiCaCl_4_^−^-***2***	*C*_*2v*_	10.92	20	−0.080	6.042	2.156	2.447	2.566	2.536	134.4
NaBeCl_4_^−^-***2***	*C*_*2v*_	0.00	22	−0.039	6.116	2.503	2.772	1.940	1.891	122.4
NaBeCl_4_^−^-***3***	*C*_*3v*_	10.36	113	0.088	5.946		2.620	2.103	1.940	115.0
NaMgCl_4_^−^-***3***	*C*_*3v*_	0.00	79	0.025	6.573		2.657	2.420	2.277	121.0
NaMgCl_4_^−^-***2***	*C*_*2v*_	4.42	21	−0.038	6.081	2.504	2.805	2.291	2.242	127.4
NaCaCl_4_^−^-***3***	*C*_*3v*_	0.00	63	0.003	6.786		2.664	2.688	2.565	125.8
NaCaCl_4_^−^-***2***	*C*_*2v*_	11.12	19	−0.040	5.998	2.509	2.804	2.570	2.534	131.8

a Cl_*t*_M for isomer MM′Cl_4_^−^-***2*** and MCl_*b*_, for MM′Cl_4_^−^-***3***.

Italics values represents that the number of bridging X atoms.

**TABLE 2:** Relative energies *E*_rel_ (kcal/mol), the lowest vibrational frequencies *υ* (cm^−1^), total NBO charges on MBr subunit (|e|), vertical detachment energies VDE (eV), bond lengths (Å), and select bond angles (degree) of the MM′Br_4_^−^ (M = Li, Na; M′ = Be, Mg, Ca) anions.

*Isomer*	Symmetry	*E*_rel_	*υ*	Q^a^	VDE	Br_*t*_-M	M-Br_*b*_	Br_*b*_-M′	M′-br_*t*_'	∠Br_*b*_M'Br_*t*_'
LiBeBr_4_^−^-***2***	*C*_*2v*_	0.00	14	−0.087	5.792	2.312	2.584	2.103	2.051	123.2
LiBeBr_4_^−^-***3***	*C*_*3v*_	7.93	77	0.060	5.795		2.452	2.275	2.089	116.8
LiMgBr_4_^−^-***3***	*C*_*3v*_	0.00	53	−0.020	6.174		2.491	2.592	2.423	123.5
LiMgBr_4_^−^-***2***	*C*_*2v*_	3.88	13	−0.089	5.750	2.313	2.616	2.448	2.398	128.5
LiCaBr_4_^−^-***3***	*C*_*3v*_	0.00	42	−0.002	6.296		2.492	2.852	2.713	128.5
LiCaBr_4_^−^-***2***	*C*_*2v*_	10.36	12	−0.100	5.650	2.317	2.609	2.721	2.690	133.1
NaBeBr_4_^−^-***2***	*C*_*2v*_	0.00	13	−0.046	5.730	2.658	2.930	2.103	2.054	122.1
NaBeBr_4_^−^-***3***	*C*_*3v*_	9.50	73	0.106	5.470		2.777	2.272	2.105	114.4
NaMgBr_4_^−^-***3***	*C*_*3v*_	0.00	50	0.032	6.080		2.817	2.587	2.434	120.1
NaMgBr_4_^−^-***2***	*C*_*2v*_	3.77	13	−0.048	5.707	2.658	2.962	2.449	2.399	126.5
NaCaBr_4_^−^-***3***	*C*_*3v*_	0.00	40	0.001	6.322		2.822	2.850	2.718	124.8
NaCaBr_4_^−^-***2***	*C*_*2v*_	10.27	12	−0.051	5.640	2.662	2.961	2.726	2.687	130.8

a Br_*t*_M for isomer MM′Br_4_^−^-***2*** and MBr_*b*_, for MM′Br _4_^−^-***3***.

Italics values represents that the number of bridging X atoms.

It can be seen in Figure 1 that the MM'Cl_4_^−^-***2*** and MM'Cl_4_^−^-***3*** isomers possess planar and three-dimensional structures with *C*_2v_ and *C*_3v_ symmetries, respectively. From the data in Table 1, it is found that for MM'Cl_4_^−^-***2*** and MM'Cl_4_^−^-***3*** isomers, when M′ atom varies from Be to Ca, the Cl_*t*_-M and M-Cl_*b*_ bond lengths change very little, while the ∠Cl_*b*_M'Cl_*t*_ angle showed a tendency of increasing; for example, the orders of ∠Cl_*b*_M'Cl_*t*_ angle in NaM'Cl_4_^−^-***2*** and NaM'Cl_4_^−^-***3*** are 122.4° < 127.4° < 131.8° and 115.0° < 121.0° < 125.8° with varying M′ atoms, respectively. Thus, the MM'Cl_4_^−^-***2*** and MM'Cl_4_^−^-***3*** structures tend to elongate along the M-M′ axis with the increasing radius of M′ atoms. Besides, the Cl_*t*_-M bond is shorter than the M-Cl_*b*_ bond in MM'Cl_4_^−^-***2*** isomers. For instance, the Cl_*t*_-Na bonds are about 2.80 Å, while the Na-Cl_*b*_ bonds are 2.50 Å in NaM'Cl_4_^−^-***2*** isomers. On the other hand, when the M atom goes from Li to Na, the Cl_*b*_-M′ and M′-Cl_*t*_’ bond lengths also show minor difference in MM'Cl_4_^−^ anions, but the ∠Cl_*b*_M'Cl_*t*_’ angles show a decrease, e.g., the ∠Cl_*b*_M'Cl_*t*_’ angles of LiBeCl_4_^−^-***2*** and LiBeCl_4_^−^-***3*** are 1.4 and 1.6° larger than that of the corresponding NaBeCl_4_^−^-***2*** and NaBeCl_4_^−^-***3***, respectively. In addition, the total NBO charges of Cl_*t*_M subunits are in the range of -0.080–0.088|e|, which are close to zero (see Table 1); consequently, the total NBO charges of M'Cl_3_ subunits approximate -1. In this sense, the MM'Cl_4_^−^ structures can be regarded as a combination of an MCl molecule and a superhalogen anion M'Cl_3_^−^.

MM'Br_4_^−^ series show similar structural characteristics with MM'Cl_4_^−^ anions. From the data in Tables 1, 2, it is noticed that ∠X_*b*_M'X_*t*_’ angles of MM'Cl_4_^−^ anions are always larger than that of the corresponding MM'Br_4_^−^ anions; for example, the ∠Cl_*b*_M'Cl_*t*_’ angles of NaMgCl_4_^−^-***3*** and NaMgCl_4_^−^-***2*** are 121.0° and 127.4°, respectively, which are larger than the ∠Br_*b*_M'Br_*t*_’ angles of NaMgBr_4_^−^-***3*** (120.1°) and NaMgBr_4_^−^-***2*** (126.5°), respectively.

It is reported that the isomers could exhibit higher stability with more bridging ligands in the previous studies on the dual-nuclear superhalogen anions with F ligands, such as homonuclear Mg_2_F_5_^−^(Anusiewicz and Skurski, 2007) and heteronuclear ones NaM'F_4_^−^(M' = Mg, Ca) (Yang et al., 2017). MM'X_4_^−^ anions also follow this rule except the MBeX_4_^−^ series. However, MBeX_4_^−^ anions show a reverse trend; that is, structure 2 is more stable than structure *3*. In other words, Be atoms are more likely to bond with three ligands than four ligands. This may be due to the smaller atomic radius of the central Be atom, the three bridging ligands are more crowded in structure 3 of MBeX_4_^−^ anions than in structure 3 of MMgX_4_^−^ and MCaX_4_^−^. This can be confirmed by the shorter Cl_b_-Cl_b_ and Br_b_-Br_b_ distance in structures 3 of MBeX_4_^−^ than MMgX_4_^−^ and MCaX_4_^−^; for example, the Cl_*b*_-Cl_*b*_ and Br_*b*_-Br_*b*_ distances in LiBeCl_4_^−^-*3* and LiBeBr_4_^−^-*3* are 0.226 Å and 0.229 Å shorter than that in LiMgCl_4_^−^-***3*** and LiMgBr_4_^−^-***3***, respectively. Thereby, MBeX_4_^−^-***3*** is less stable than the MBeX_4_^−^-***2***.

### Vertical Electron Detachment Energies (VDEs)

The VDE values of MM'X_4_^−^ anions are gathered in Tables 1, 2. As one can notice, all anions have considerable VDE values (5.470–6.799 eV) exceeding the electron affinity of the Cl atom; thus undoubtedly, these anions can be identified as superhalogen anions. In addition, it is found that the factors affecting the VDE values of these studied anions were as follows:

(1) When the M atom varies from the Li atom to Na atom, the VDE values of the studied anions show a tendency of decreasing in similar structures. For example, the VDE values of the isomers LiMgCl_4_^−^-***3*** and LiMgCl_4_^−^-***2*** are greater than those of NaMgCl_4_^−^-***3*** and NaMgCl_4_^−^-***2***, respectively. However, the only one exception is the VDE values of isomers LiCaBr_4_^−^-***3*** and NaCaBr_4_^−^-***3*.** This is probably due to the different extra electron distribution of these two isomers, which will be discussed in the following. Therefore, the hetero-binuclear superhalogen anions with large VDE values could be constructed by introducing small alkali metal atoms into the system. It is worth noting that the same trend was found for the other hetero-binuclear anions with the F atom, cyanide, and isocyanide as ligands (Yang et al., 2017; Yang et al., 2018).
(2) The largest VDE values for each MM'X_4_^−^ anions are presented in Table 3. From the table, the VDE values increase in the order: MBeX_4_^−^→MMgX_4_^−^→MCaX_4_^−^. Hence, the hetero-binuclear superhalogen anion MM'X_4_^−^ could possess a larger VDE value by involving larger alkaline earth metal atoms. Note that it also holds true for the superhalogen anions with other ligands (Yang et al., 2017; Yang et al., 2018).

**TABLE 3:** The largest vertical detachment energies VDE (eV) of superhalogen anions MM′X_4_^−^ (M = Li, Na; M′ = Be, Mg, Ca, X = Cl, Br) and M′X_3_^−^ (M′ = Be, Mg, Ca, X = Cl, Br).

*anion*	VDE	*anion*	VDE	*anion*	VDE
LiBeCl_4_^−^	6.275	NaBeCl_4_^−^	6.116	BeCl_3_^−^	6.184
LiMgCl_4_^−^	6.700	NaMgCl_4_^−^	6.573	MgCl_3_^−^	6.685
LiCaCl_4_^−^	6.799	NaCaCl_4_^−^	6.786	CaCl_3_^−^	6.741
LiBeBr_4_^−^	5.795	NaBeBr_4_^−^	5.730	BeBr_3_^−^	5.643
LiMgBr_4_^−^	6.174	NaMgBr_4_^−^	6.080	MgBr_3_^−^	6.140
LiCaBr_4_^−^	6.296	NaCaBr_4_^−^	6.322	CaBr_3_^−^	6.243

As pointed out earlier, the MM'X_4_^−^ anions can be regarded as MX (M'X_3_)^−^; thus, the comparison between MM'X_4_^−^ anions and their corresponding mononuclear superhalogen anions M'X_3_^−^ is also necessary. For this reason, the VDE values of M'X_3_^−^ (X = Cl, Br) anions were also calculated at the same level and are listed in Table 3 as well. From the table, the VDE values of mononuclear anions M'X_3_^−^ also increase from BeX_3_^−^ to CaX_3_^−^. Besides, the mononuclear anions M'X_3_^−^ possess lower VDE values than their corresponding hetero-nuclear anions MM'X_4_^−^ (except for NaBeCl_4_^−^ and NaMgX_4_^−^ series). So again, the superhalogen anions could gain larger VDE values by increasing the number of central atoms.

**FIGURE 2:**
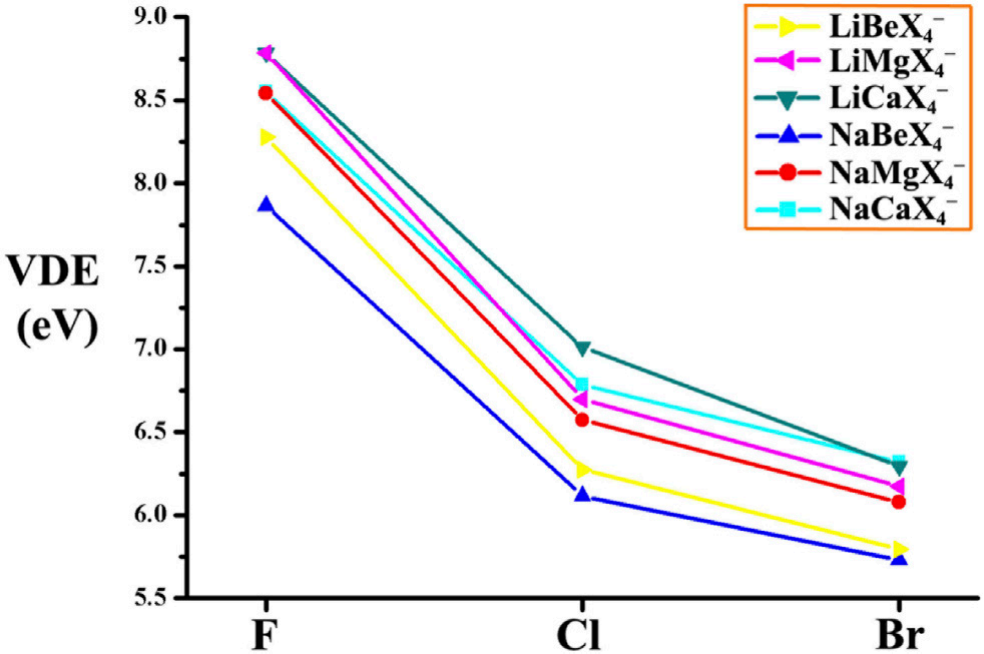
The evolutions of VDE values of the MM′X_4_^−^ and M′X_3_^−^ (M = Li, Na; M′ = Be, Mg, Ca, X = F ((from ref. [4]), Cl, Br) anions.

**FIGURE 3:**
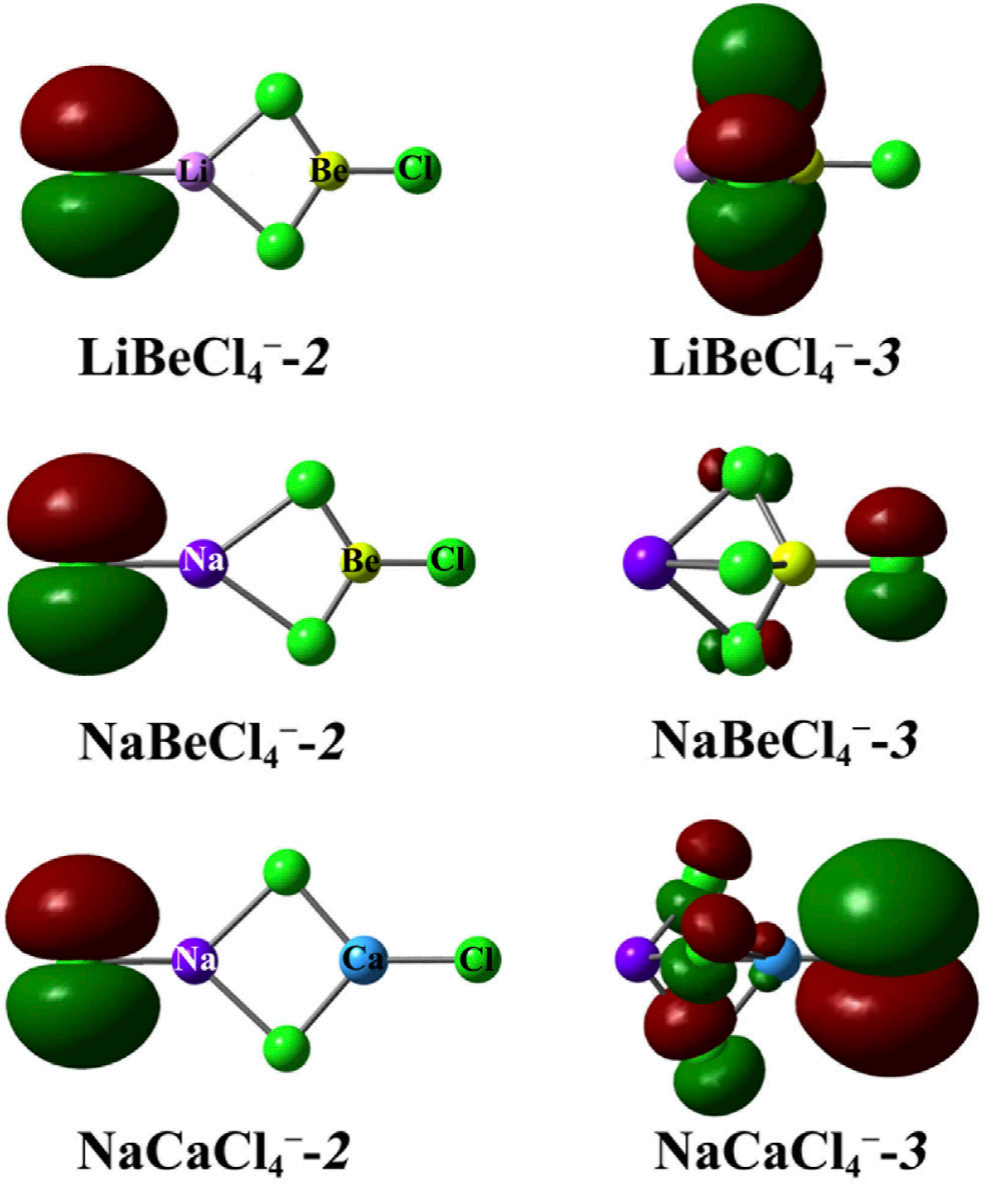
HOMO orbitals of superhalogen anions LiBeCl_4_^−^, NaBeCl_4_^−^, and NaCaCl_4_^−^.Color legend: green for Cl atom, light purple for Li atom, dark purple for Na atom, and blue for Ca atom.

(3) The relationship between the VDE values and the ligand atoms is plotted in Figure 2. The six curves show similar varying trends, that is, the largest VDE values of each MM'X_4_^−^ (X = F (Yang et al., 2017), Cl, Br) species show a decreasing order: MM'F_4_^−^ > MM'Cl_4_^−^ > MM'Br_4_^−^. This may be attributed to the different electronegativity of X atoms. To be specific, the F atom possesses larger electronegativity and stronger electron-accepting ability than Cl and Br atoms, which is more beneficial for the anions to bind with the extra electron. Thereby, the larger electronegativity the ligand atom possesses, the higher VDE value the MM'X_4_^−^ anion has.
(4) For the two isomers of LiM'X_4_^−^, the VDE values of LiM'X_4_^−^-***3*** are always larger than those of LiM'X_4_^−^-*2*. This is probably due to the fact that the extra electron distribution in two isomers is different. To analyze this clearly, the highest occupied molecular orbitals (HOMOs) of some representative MM'X_4_^−^ isomers are depicted in Figure 3. As can be seen from the figure, the extra electron is confined to a single X_*t*_ atom in LiBeCl_4_^−^-***2***, while localized on the three bridging X_*b*_ atoms in LiBeCl_4_^—^ ***3,*** which is a benefit for the extra negative charge dispersion, and thus, LiBeCl_4_^−^-***3*** possesses a larger VDE value. For NaBeX_4_^−^ anions, the extra electrons of two isomers are all distributed on the terminal X atom. Interestingly, the isomer NaBeX_4_^−^-***2*** in which the extra electron goes on X_*t*_ atom has a higher VDE value than isomer NaBeX_4_^—^ ***3*** *,* which goes on the X_*t*_’ atom (see Figure 3). As to NaMgX_4_^−^ and NaCaX_4_^−^ species, the situation is similar to that of the LiM'X_4_^−^ anions. However, unlike LiM'X_4_^−^-***3*** and NaMgX_4_^−^-***3***, the extra electron is shared by all X ligand atoms instead of three X_*b*_ ligands in NaCaX_4_^−^-***3***, which leads to the extra negative charge being more evenly distributed (see Figure 3), and, hence, a relatively larger VDE values for these isomers. This may also explain why NaCaBr_4_^−^-***3*** exhibits larger VDE values than LiCaBr_4_^−^-***3.*** Therefore, the extra electron distribution is an important factor affecting the VDE values of the hetero-binuclear superhalogen anions.

## Conclusion

Our systematic investigation of the MM′X_4_^−^ (M = Li, Na; M′ = Be, Mg, Ca; X = Cl, Br) species has theoretically proposed a series of hetero-binuclear superhalogen anions. The results show that these heteronuclear superhalogen anions could gain larger VDE values by involving a smaller alkali metal atom M, a larger alkaline earth metal atom M′, and a higher electronegative ligand atom X. Thereby, of all the anions studied, an isomer of LiCaCl_4_^−^ anions possesses the largest VDE value (6.799 eV). Moreover, the extra electron distribution is a very influential factor in the VDE values of structural isomers. For the NaBeX_4_^−^ anions, the isomers have larger VDE values when the extra electron is distributed on the terminal X_*t*_ ligand atom instead of the X_*t*_’ ligand atom. For the other anions, the isomers possess larger VDE values when the extra electron is shared by all ligand atoms or three bridging ligand atoms.

## Data Availability

The original contributions presented in the study are included in the article/supplementary material; further inquiries can be directed to the corresponding authors.
